# More than random responding: Empirical evidence for the validity of the (Extended) Crosswise Model

**DOI:** 10.3758/s13428-022-01819-2

**Published:** 2022-04-21

**Authors:** Julia Meisters, Adrian Hoffmann, Jochen Musch

**Affiliations:** Department of Experimental Psychology, University of Duesseldorf, Universitaetsstrasse 1, 40225 Duesseldorf, Germany

**Keywords:** Randomized response technique, Extended crosswise model, Random responding, Validity

## Abstract

The Randomized Response Technique (Warner, *Journal of the American Statistical Association*, *60*, 63-69, 1965) has been developed to control for socially desirable responses in surveys on sensitive attributes. The Crosswise Model (CWM; Yu et al., *Metrika, 67*, 251-263, 2008) and its extension, the Extended Crosswise Model (ECWM; Heck et al., *Behavior Research Methods, 50,* 1895-1905, 2018), are advancements of the Randomized Response Technique that have provided promising results in terms of improved validity of the obtained prevalence estimates compared to estimates based on conventional direct questions. However, recent studies have raised the question as to whether these promising results might have been primarily driven by a methodological artifact in terms of random responses rather than a successful control of socially desirable responding. The current study was designed to disentangle the influence of successful control of socially desirable responding and random answer behavior on the validity of (E)CWM estimates. To this end, we orthogonally manipulated the direction of social desirability (undesirable vs. desirable) and the prevalence (high vs. low) of sensitive attributes. Our results generally support the notion that the ECWM successfully controls social desirability bias and is inconsistent with the alternative account that ECWM estimates are distorted by a substantial influence of random responding. The results do not rule out a small proportion of random answers, especially when socially undesirable attributes with high prevalence are studied, or when high randomization probabilities are applied. Our results however do rule out that random responding is a major factor that can account for the findings attesting to the improved validity of (E)CWM as compared with DQ estimates.

In surveys on sensitive topics, the validity of prevalence estimates obtained via direct self-reports is threatened by social desirability bias when respondents choose to reply in line with social or legal norms rather than truthfully (Paulhus, 1991; Tourangeau & Yan, 2007). To increase respondents’ motivation to answer honestly, and thus control for the influence of social desirability on self-reports, the Randomized Response Technique was developed (RRT; Warner, 1965). The general idea behind the RRT is to add random noise to respondents’ answers employing a randomization procedure, thereby maximizing the confidentiality of individual answers. For example, respondents could be instructed to roll a die in secret, and then answer “true” or “false” to either a sensitive statement A if they rolled a number between 1 and 4 (e.g., “I have consumed cocaine”), or to its negation B if they rolled a 5 or 6 (e.g., “I have *never* consumed cocaine”). As the outcome of the die roll remains unknown to the experimenter, it is impossible to tell whether an individual respondent answered statement A or statement B, thus eliminating any link between their answer and their status with respect to the sensitive attribute. Utilizing the known distribution of randomization outcomes, however, allows estimating the prevalence of the sensitive attribute on sample level. If the confidentiality protection offered by the RRT indeed increases the validity of self-reports by controlling for socially desirable responses, prevalence estimates obtained via the RRT are expected to be higher than prevalence estimates obtained via direct questioning (DQ) for socially undesirable attributes (“more is better”) and lower than prevalence estimates obtained via DQ for socially desirable attributes (“less is better”). A meta-analysis of 32 comparative validation studies found that the RRT resulted in higher, and thus presumably more valid, prevalence estimates than DQ (Lensvelt-Mulders et al., 2005). Results from such comparative validations however only offer “weak” evidence for the method’s validity, as the true prevalence of the sensitive attribute remains unknown, and the prevalence estimates obtained could thus still be overestimates or underestimates (Moshagen et al., 2014; Umesh & Peterson, 1991). The same meta-analysis also included six so-called “strong” validation studies in which the prevalence of the sensitive attribute was known and could be compared to the estimates obtained. This analysis revealed that while RRT estimates were substantially closer to the true value than DQ estimates, the RRT still underestimated the true prevalence, and its validity was therefore imperfect (Lensvelt-Mulders et al., 2005). To overcome some of the drawbacks of the original RRT, several advancements of the method have been proposed (for an overview see, e.g., Antonak & Livneh, 1995; Cerri et al., 2021; Chaudhuri, 2011; Chaudhuri & Christofides, 2013; Fox & Tracy, 1980, 1986; Franklin, 1998; Greenberg et al., 1974; Horvitz et al., 1976; Scheers, 1992; Tracy & Mangat, 1996).

## The Crosswise Model

**Fig. 1:**
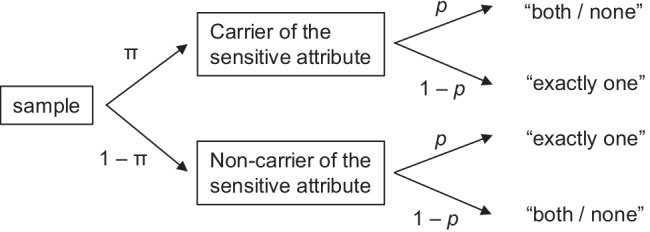
Tree diagram of the Crosswise Model. The parameter π represents the unknown prevalence of the sensitive attribute, which has to be estimated; *p* represents the known randomization probability

The Crosswise Model (CWM; Yu et al., 2008) is a recent advancement of the RRT that has steadily gained popularity among empirical researchers over the past decade. In the CWM, respondents are presented with two statements simultaneously: a sensitive statement A for which the prevalence π is to be estimated (e.g., “I have consumed cocaine”), and a non-sensitive statement B that is used for randomization with known prevalence *p* (e.g., “I was born in November or December”). Respondents are asked to provide a joint answer by indicating whether they agree with “*both* statements or *none* of the statements” or whether they agree with “*exactly one* of the statements (irrespective of which one)”. Notably, neither of these response options reveals whether an individual respondent is a carrier of the sensitive attribute or not, thus granting full confidentiality of individual answers. However, since the prevalence *p* of the non-sensitive attribute is known (e.g., from official birthing statistics), a maximum likelihood estimate for the prevalence π of the sensitive attribute on sample level can be obtained by using the formula (Yu et al., 2008):

1 $${\hat{\uppi}}_{\mathrm{CWM}}=\frac{\frac{n^{\prime }}{n}+\mathrm{p}-1}{2\ast \mathrm{p}-1},p\ne \frac{1}{2}$$

with *n*^′^representing the total number of “both/ none” responses and *n* being the sample size. Figure 1 shows the CWM as a tree diagram. In contrast to the original RRT, the CWM does not require an external randomization device and is thus easier to apply for both respondents and experimenters (Yu et al., 2008). Moreover, the CWM has been shown to provide higher levels of comprehension, and perceived confidentiality protection, than other RRT variants (Hoffmann et al., 2017). In contrast to many other RRT variants, the CWM offers the specific advantage of “response symmetry”, meaning that none of the answer options is a “safe” alternative that respondents following a self-protective strategy could choose to explicitly deny being a carrier of the sensitive attribute. Presumably due to this favorable property, the CWM has recently been found to be robust towards deliberate positive self-presentation (Hoffmann et al., 2021).

The validity of the CWM has been investigated in many comparative validation studies. Many of those studies found “weak” evidence for the method’s validity employing the “more is better” criterion, since prevalence estimates obtained via the CWM were usually higher than prevalence estimates obtained via DQ for socially undesirable attributes such as xenophobia (Hoffmann et al., 2020; Hoffmann & Musch, 2016; Meisters et al., 2020b), the use of anabolic steroids among bodybuilders (Nakhaee et al., 2013), distrust in the Trust Game (Thielmann et al., 2016), plagiarism (Jann et al., 2012), tax evasion (Korndörfer et al., 2014; Kundt et al., 2017), prejudice against female leaders (Hoffmann & Musch, 2019), crossing the street on a “Don’t Walk” sign in plain view of children (Hoffmann et al., 2021), and the intention to vote for the German right-wing party *Alternative for Germany* (Waubert de Puiseau et al., 2017). For the socially desirable attribute of washing hands during the COVID-19 pandemic, CWM estimates were significantly lower than prevalence estimates obtained via DQ and thus also presumably more valid according to the “less is better” criterion (Mieth et al., 2021).

The two most recent meta-analyses on weak validation studies have also shown that the CWM leads to higher prevalence estimates for socially undesirable and lower prevalence estimates for socially desirable attributes than DQ, with the advantage of the CWM being more pronounced for more sensitive topics and more educated respondents (Sagoe et al., 2021; Schnell & Thomas, 2021). Furthermore, “strong” validation studies have shown that the CWM correctly estimated the known prevalence of experimentally induced cheating behavior (Hoffmann et al., 2015) as well as the prevalence of a non-sensitive control attribute (Hoffmann et al., 2020; Hoffmann & Musch, 2016, 2019). However, in some studies, the CWM provided prevalence estimates that did not differ from DQ estimates or were even negative (Hoffmann et al., 2020; Höglinger et al., 2016; Jerke et al., 2022). Moreover, in recent studies with a known status of individual respondents the CWM was found to sometimes produce false positives because some non-carriers of the sensitive attribute were falsely classified as carriers. False positives might lead to problematic overestimations, especially when the true prevalence is close to zero, but can be reduced using detailed instructions and comprehension checks for highly educated respondents (Höglinger & Diekmann, 2017;Höglinger & Jann, 2018 ; Meisters et al., 2020a).

It is not yet fully understood what may explain the mixed results regarding the validity of the CWM. One potential explanation for the mixed findings might be found in the different types of attributes under investigation. For example, in some studies that found false positives in the CWM, attributes with a prevalence of zero were assessed (e.g., Höglinger & Diekmann, 2017). Using attributes that clearly do not apply to respondents might be problematic because zero-prevalence attributes could lead respondents to perceive the CWM method or the entire survey as pointless, potentially resulting in low motivation to comply with the instructions, or even in deliberate response biases. Furthermore, zero-prevalence attributes have the specific disadvantage that they do not allow the assessment of false negatives because by definition, no carriers of the sensitive attribute are present if the prevalence is zero. Deviations from a true prevalence of zero can therefore only occur in the form of false positives, and false negatives can never be observed.

Other studies that found false positives in the CWM used experimentally induced sensitive attributes for which the prevalence could be determined within the sample, and was well above zero (Höglinger & Jann, 2018; Meisters et al., 2020a). While this approach avoids some of the problems associated with zero-prevalence attributes, experimentally induced attributes might also be problematic due to a potential self-deception of respondents. For example, some respondents who are classified as “cheaters” in a given task because they overreported their performance could actually deceive themselves into thinking their self-reports were accurate. If these respondents are then questioned about their cheating behavior with a CWM question, they might honestly, but falsely, deny being a carrier of the sensitive attribute. This kind of self-deception cannot be overcome by any currently established indirect questioning technique.

Another potential explanation for the mixed results regarding the validity of the CWM could be found in an insufficient comprehension of the instructions. There is evidence that while the CWM is more comprehensible than other RRTs (Hoffmann et al., 2017), the cognitive burden it places on the respondents may still be too high. For example, many respondents failed comprehension questions for the CWM, and this problem was particularly pronounced among lower-educated respondents (Meisters et al., 2020a). Moreover, some respondents have been found to answer carelessly or inattentively (Atsusaka & Stevenson, 2021). Both cognitive overload and general inattentiveness might potentially result in random responses (Atsusaka & Stevenson, 2021; Höglinger & Diekmann, 2017; Walzenbach & Hinz, 2019). Indeed, when directly queried about their response behavior, between 2 and 19% of the respondents admitted to having randomly selected one of the response options in the CWM (Enzmann, 2017; Meisters et al., 2020a; Schnapp, 2019). Such estimates of random responding based on direct self-reports should generally be interpreted with caution, since they refer to a potentially sensitive behavior and might themselves be subject to the influence of social desirability bias, or careless responding. Yet, a substantial share of random responses would have severe consequences for the validity of CWM estimates.

## A potentially problematic influence of random responding

First, random responses could result in false positives and false negatives in CWM applications (Höglinger & Diekmann, 2017; Höglinger & Jann, 2018; Meisters et al., 2020a). False positives as a consequence of random responding would be especially likely for attributes with a prevalence close to zero since in this case, any proportion of random responses would lead to some non-carriers being falsely classified as carriers. Moreover, random responding in the CWM could reduce the observed effect of potential covariates due to an increase in statistical noise (Enzmann, 2017). The most serious problem, however, is that random responding biases prevalence estimates obtained via the CWM towards 50% (Walzenbach & Hinz, 2019). Such a bias is especially problematic in comparative validation studies investigating socially undesirable attributes with a prevalence of less than 50% and relying on the “more is better” criterion (as most previous validation studies, e.g. Hoffmann & Musch, 2016, 2019; Jann et al., 2012; Korndörfer et al., 2014; Thielmann et al., 2016; Waubert de Puiseau et al., 2017), and in studies investigating socially desirable attributes with a prevalence of more than 50% and relying on the “less is better” criterion (e.g. Mieth et al., 2021). In such studies, estimates for undesirable attributes that were higher in a CWM compared to a DQ condition (“more is better”), or estimates for desirable attributes that were lower in a CWM than in a DQ condition (“less is better”) have repeatedly been interpreted as evidence for a successful control of social desirability bias. An alternative account for the same findings is however the occurrence of a methodological artifact resulting from a substantial share of random responses in the CWM condition that biases estimates towards 50% (Walzenbach & Hinz, 2019). As both a successful control of social desirability and random responses in CWM surveys would influence prevalence estimates in the same direction (that is, towards 50%), no previous comparative validation study allows disentangling these two alternative accounts (Walzenbach & Hinz, 2019).

Based on this observation, Walzenbach and Hinz (2019) have proposed a novel framework for comparative validation studies that allows separating the influence of a successful control of social desirability bias and random responding. The basic idea of this validation approach is to combine the “more is better” and the “less is better” criterion by investigating sensitive attributes from each of the four possible combinations resulting from crossing the direction of social desirability (socially undesirable vs. socially desirable) and the prevalence of the respective attribute (below 50% vs. above 50%). For example, they proposed assessing the attributes of blood donation as a socially desirable attribute with a prevalence below 50% and passing a red traffic light as a socially undesirable attribute with a prevalence above 50%. A comprehensive comparison of CWM and DQ prevalence estimates in all four possible cells allows to test two competing hypotheses: If the CWM indeed controls for the influence of socially desirable responding, CWM estimates should consistently be higher than DQ estimates for socially undesirable attributes, and lower than DQ estimates for socially desirable attributes, irrespective of overall prevalence. If, however, CWM estimates are predominantly influenced by random responding, they should consistently be higher than DQ estimates for attributes with a prevalence below 50%, and lower than DQ estimates for attributes with a prevalence above 50%, irrespective of the direction of social desirability. The most interesting comparisons that allow going beyond previous comparative validations can be found in socially undesirable attributes with a prevalence above 50%, and socially desirable attributes with a prevalence below 50%. For such attributes, the successful control of social desirability bias and random responses influence prevalence estimates in opposite directions. Consequently, they allow for the most rigorous test of the two opposing accounts for previous findings obtained using the CWM.

In the first study using this framework, the prevalence estimate for a socially undesirable attribute with low prevalence (blood donation) obtained via the CWM was descriptively higher than the prevalence estimate obtained via DQ (Walzenbach & Hinz, 2019). While inferential statistics for the comparison of the estimates were not reported, this pattern of results was more in line with random responses biasing the CWM estimate than with a successful control of social desirability bias. However, this study was not initially designed to test the differential influence of control for social desirability versus random responding, and therefore only one of the four possible combinations of the prevalence (low vs. high) and direction of social desirability (undesirable vs. desirable) was investigated. For a comprehensive evaluation based on the framework proposed by Walzenbach and Hinz (2019), it is essential to compare CWM and DQ prevalence estimates for sensitive attributes from all four possible combinations of prevalence (low vs. high) and direction of social desirability (undesirable vs. desirable) in an experimental setting. To conduct such a comparison was the aim of the present study.

## The present study

The present study is the first to apply all four cells from the framework proposed by Walzenbach and Hinz (2019) to disentangle the differential influence of a successful control of social desirability bias versus random responding. To achieve this goal, we experimentally and orthogonally manipulated the direction of social desirability (socially undesirable vs. socially desirable), the prevalence of the sensitive attribute (low, i.e. below 50%, vs. high, i.e. above 50%), and the questioning technique applied (DQ vs. the Extended Crosswise Model). Importantly, we assessed real sensitive attributes with a prevalence well above zero, thus avoiding problems potentially associated with zero-prevalence items or experimentally induced sensitive attributes. As an indirect questioning technique, we opted for the Extended Crosswise Model (ECWM; Heck et al., 2018), a recent advancement of the original CWM. The format of ECWM questions and answer options is identical to the format of the original CWM; however, in the ECWM design, two non-overlapping groups of respondents are confronted with questions including the same sensitive statement, but different non-sensitive statements used for randomization. The non-sensitive statements are chosen to result in different randomization probabilities *p1* and *p2* in the two groups (e.g., *p1* = 1 - *p2*). This simple extension provides the ECWM with one degree of freedom and thus allows assessing the fit of the model to empirically observed data. If the model fit is acceptable, prevalence estimates from the two ECWM groups can be pooled into a single estimate; if, however, a model misfit is observed, the pooled estimate is potentially subject to the influence of response biases and should be interpreted with caution. The ECWM retains all advantages of its predecessor, the CWM, including its high statistical efficiency, but adds the specific advantage of the possibility to test the fit of the model. Importantly, however, the ECWM can only detect systematic response biases (Heck et al., 2018) and cannot detect random answer biases, as random responding would bias ECWM estimates just as it would bias CWM estimates (that is, towards 50%). Random responding could however be detected in the present study because all four of the cells that are relevant to conclude the presence of random answers were part of our experimental design.

Two previous studies have compared ECWM to DQ estimates for the prevalence of a socially undesirable attribute with low prevalence (Meisters et al., 2020b) and a socially desirable attribute with high prevalence (Mieth et al., 2021). None of these studies however allowed to disentangle the influence of a successful control of social desirability and random responses because, in each study, only one of the four relevant cells that were outlined above was part of the respective experimental design.

Based on our experimental design, we expected to observe one of the following two patterns of results (cf. Table 1): If irrespective of their prevalence (low vs. high), ECWM estimates are higher than DQ estimates for socially undesirable (“more is better”), and lower than DQ estimates for socially desirable attributes (“less is better”), this would lend strong support to the notion that the difference between ECWM and DQ estimates can primarily be ascribed to the successful control of social desirability bias by the ECWM, rather than a methodological artifact due to random responding. If, however, ECWM estimates are higher than DQ estimates for sensitive attributes with low prevalence (< 50%) and lower than DQ estimates for sensitive attributes with high prevalence (> 50%), irrespective of the direction of social desirability (undesirable vs. desirable), the difference between ECWM and DQ prevalence can better be explained by a problematic influence of random responding biasing the ECWM estimate towards 50%, rather than a successful control of social desirability. Of particular relevance in deciding in favor of one of the two opposing explanatory approaches are the attributes for which the two potential influencing variables exert an opposite influence – that is, socially undesirable attributes with a high prevalence and socially desirable attributes with a low prevalence. For this reason, unlike in previous studies, these two cells were both parts of the experimental design used in the present study.

**Table 1:** Result patterns that are to be expected for prevalence estimates of sensitive attributes a) if the ECWM provides a successful control of social desirability, and b) if the ECWM profits from a statistical bias due to random responses

	Successful control of social desirability		Bias due to random responses	
	Low prevalence (< 50%)	High prevalence (> 50%)	Low prevalence (< 50%)	High prevalence (> 50%)
Socially undesirable	$\hat{\uppi}$_ECWM_ > $\hat{\uppi}$_DQ_	$\hat{\boldsymbol{\uppi}}$_**ECWM**_ **>** $\hat{\boldsymbol{\uppi}}$_**DQ**_	$\hat{\uppi}$_ECWM_ > $\hat{\uppi}$_DQ_	$\hat{\boldsymbol{\uppi}}$_**ECWM**_ **<** $\hat{\boldsymbol{\uppi}}$_**DQ**_
Socially desirable	$\hat{\boldsymbol{\uppi}}$_**ECWM**_ **<** $\hat{\boldsymbol{\uppi}}$_**DQ**_	$\hat{\uppi}$_ECWM_ < $\hat{\uppi}$_DQ_	$\hat{\boldsymbol{\uppi}}$_**ECWM**_ **>** $\hat{\boldsymbol{\uppi}}$_**DQ**_	$\hat{\uppi}$_ECWM_ < $\hat{\uppi}$_DQ_

DQ = Direct Questioning, ECWM = Extended Crosswise Model. *Bold print* marks the cells in which a different pattern of results is to be expected depending on whether the ECWM allows controlling for social desirability or whether ECWM estimates are distorted by random responses

## Method

## Participants

The initial sample consisted of *N* = 7172 adult native German speakers registered with a German commercial online panel. Of the initial respondents, *n* = 668 (9.31%) dropped out before completing the questionnaire. The dropout rate differed significantly between the two questioning technique conditions, DQ: 1.93%, ECWM: 12.65%, χ*²*(1) = 209.37, *p* <.001, *Cramer’s V* = .17. The final sample consisted of *N* = 6504 respondents (50.14% male, 49.68% female, 0.18% diverse). The distribution of respondents across the conditions can be found in Table 2. The exact distribution of age and education on the questioning techniques can be found in Table 3.

**Table 2:** Distribution of respondents across experimental conditions

		Questioning technique condition			
Social desirability condition	Prevalence condition	ECWM p1	ECWM p2	DQ	Sum
Socially undesirable	Low prevalence	538 (8.27%)	535 (8.23%)	545 (8.38%)	1618 (24.88%)
	High prevalence	541 (8.32%)	538 (8.27%)	553 (8.50%)	1632 (25.09%)
Socially desirable	Low prevalence	545 (8.38%)	535 (8.23%)	549 (8.44%)	1629 (25.05%)
	High prevalence	543 (8.35%)	540 (8.30%)	542 (8.33%)	1625 (24.98%)
Sum		2167 (33.32%)	2148 (33.03%)	2189 (33.66%)	6504 (100.00%)

DQ = Direct Questioning, ECWM *p1* = Extended Crosswise Model with randomization probability *p1*, ECWM *p2* = Extended Crosswise Model with randomization probability *p2*

**Table 3:** Demographics by questioning technique

	DQ	ECWM	
	(%)	(%)	
Gender			χ*²*(2) = 0.12, *p* = .944, *Cramer’s V* < .01
Female	49.99	50.43	
Male	49.83	49.38	
Diverse	0.19	0.18	
Age (years)			χ*²*(5) = 16.53, *p* = .005, *Cramer’s V* =.05
18–25	12.29	12.44	
26–35	21.24	22.87	
36–45	16.08	18.01	
46–55	16.63	16.22	
56–65	17.36	17.40	
> 65	16.40	13.05	
Educational achievement			χ*²*(7) = 6.46, *p* = .488, *Cramer’s V* = .03
No school leaving certificate	0.59	0.63	
Lower secondary school leaving certificate	20.24	19.10	
Secondary school leaving certificate	29.05	30.38	
Subject-specific university entrance qualification	7.40	7.97	
Higher education entrance qualification	15.62	16.36	
Bachelor’s degree	8.77	8.51	
Master’s degree	17.23	15.69	
PhD	1.05	1.38	

DQ = Direct Questioning, ECWM = Extended Crosswise Model

The survey was carried out in accordance with the revised Declaration of Helsinki (World Medical Association, 2013) and the ethical guidelines of the German Society for Psychology (Berufsverband Deutscher Psychologinnen und Psychologen & Deutsche Gesellschaft für Psychologie, 2016). In Germany, there is no binding obligation that research projects can only be carried out after approval by an ethics committee. Participation in the present study could not have any negative consequences for the respondents, and anonymity was ensured at all times. The respondents participated voluntarily and after informed consent was obtained. There was no risk that participation could cause any physical or mental damage or discomfort to participants beyond their normal everyday experiences. Therefore, ethics committee approval was not required according to the “Ethical Research Principles and Test Methods in the Social and Economic Sciences” formulated by the Ethics Research Working Group of the German Data Forum (RatSWD, 2017) and the “Ethical Recommendations of the German Psychological Society” (DGPs, 2018). The sample size was determined based on a priori power considerations (Ulrich et al., 2012) indicating that a sample of more than 6000 respondents would ensure sufficient statistical power (1-ß ≥ .80) for the planned prevalence comparisons. Twice as many respondents were allocated to the ECWM conditions compared to the DQ conditions to compensate for the generally lower efficiency of indirect questioning techniques which is a consequence of the randomization procedure (Moshagen et al., 2012; Ulrich et al., 2012).

## Survey design

Each respondent received two sensitive questions in either the ECWM *p1*, ECWM *p2,* or the DQ format that belonged to either of the four groups: Socially undesirable, low prevalence; socially undesirable, high prevalence; socially desirable, low prevalence; socially desirable, high prevalence. Respondents were randomly allocated to the conditions.

### Sensitive attributes

To identify suitable sensitive attributes covering all four possible cases (socially undesirable vs. desirable and low vs. high prevalence), we conducted a pilot study comprising *N* = 1059 adult native German speakers (50.24% male, 49.54% female, 0.19% diverse) recruited via the same German commercial online panel provider as in the main study. In the pilot study, respondents were presented with 72 presumably sensitive statements and were randomly assigned to one of two experimental conditions. In a first condition (*n* = 534, 50.42% of the sample), they were asked to indicate whether they agreed with the statements or not. Based on responses from this condition, rough estimates for the prevalence of each sensitive attribute were determined. Using DQ to obtain rough prevalence estimates in the pilot study may seem odd at first because such estimates are likely to be influenced by social desirability bias. However, the design of our main study did not require knowledge of the true prevalence of the selected attributes; instead, DQ prevalence estimates only had to be likely low (< 50%) or high (> 50%), so that these estimates could serve as a reference against which ECWM estimates could be compared. In a second condition in the pilot study, respondents were asked to rate the statements regarding their social desirability (*n* = 525, 49.58% of the sample) on an 11-point Likert scale ranging from 1 *(very socially undesirable [negative])* via 6 *[neutral]* to 11 *(very desirable [positive]).* Mean values on this scale served as a proxy for the assumed direction and strength of the influence of social desirability on each attribute. The attributes shown in Table 4 were deemed most suitable and therefore selected for the main study. To improve the generalizability of our results, we selected two attributes for each combination of direction of social desirability (undesirable vs. desirable) and prevalence (low, i.e. < 50%, vs. high, i.e. > 50%). Detailed descriptive statistics for the prevalence and social desirability ratings for the attributes chosen for the main study can be obtained from Table A1 in the Appendix on osf (https://osf.io/jer5h/).

**Table 4:** Wording of sensitive and non-sensitive statements

Social Desirability	Prevalence (low / high)	Sensitive statement	Paired non-sensitive statement
Socially undesirable	Low prevalence	I have not returned a borrowed item.	I was born in November or December.
		I have illegally disposed of trash.	My mother was born in November or December.
	High prevalence	I sometimes lie.	I was born in November or December.
		I have kept change that was mistakenly given back to me in excess.	My mother was born in November or December.
Socially desirable	Low prevalence	I have actively intervened to prevent violence against women or children.	I was born in November or December.
		I volunteer in the social sector.	My mother was born in November or December.
	High prevalence	I cast my vote in the last federal election.	I was born in November or December.
		I go for regular medical checkups.	My mother was born in November or December.

#### Questioning techniques

In the DQ conditions, respondents were presented with a sensitive statement, such as “I have illegally disposed of trash” and had to indicate whether they agreed with this statement or not. In the ECWM conditions with randomization probability *p1*, respondents were simultaneously presented with a sensitive statement, such as “I have illegally disposed of trash” and a non-sensitive statement, such as “I was born in November or December” or “My mother was born in November or December” (*p1* = .158; Pötzsch, 2012). They were asked to provide a joint answer by choosing one of the two answer options “I agree with *both* statements or I agree with *none* of the statements” versus “I agree with *exactly one* of the statements (irrespective of which one)”. In the ECWM conditions with randomization probability *p2*, the non-sensitive statements read “I was born between January and October” or “My mother was born between January and October” (*p2* = .842).

#### Self-reports of random responding

To directly assess random responding, respondents were asked to respond to the statement “I just randomly ticked one of the answers” on a 7-point Likert scale ranging from 1 (*strongly disagree*) to 7 (*strongly agree*) (cf. Meisters et al., 2022).

### Procedure

Respondents received a short introduction, provided informed consent, and were asked to answer demographic questions about their gender, age, native language, and highest school leaving qualification. Subsequently, respondents were presented with the two sensitive questions of the respective condition in either the DQ or the ECWM format. Both the order of the two sensitive questions as well as the answer options for each sensitive question were presented in randomized order. In the ECWM conditions, the questioning technique was explained in detail and respondents had to answer four comprehension questions to ensure they had understood the procedure (cf. Meisters et al., 2020a). Towards the end of the questionnaire, after having received the sensitive questions, respondents were asked to provide direct information on whether they had responded randomly. Respondents in the DQ condition were additionally asked to indicate whether they agreed with the two non-sensitive control attributes “I was born in November or December” and “My mother was born in November or December”. Finally, all respondents were thanked and debriefed.

### Statistical analyses

For our analyses, we used R (version 4.0.5; R Core Team, 2021) and the package RRreg (version 0.6.2; Heck & Moshagen, 2018). For each of the eight sensitive attributes and each questioning technique group (DQ, ECWM *p1*, ECWM *p2*), parameter estimates for the prevalence π were derived based on the empirically observed answer frequencies. The model fit of the ECWM was then assessed separately for each sensitive attribute via the asymptotically Chi²-distributed log-likelihood statistic *G²*. To this end, we tested the fit of an overall ECWM model in which the prevalence parameters from the two ECWM groups were equalized and pooled into a single parameter (π_ECWM_1_ = π_ECWM_2_ = π_ECWM_), resulting in a model with one degree of freedom. If this overall model fit the data well (*p* ≥ .05), the pooled ECWM estimate was considered trustworthy; in case of an insufficient model fit (*p* < .05), the pooled ECWM estimate was considered to be potentially biased, and additional analyses using estimates from the two ECWM groups as lower and upper bound estimates were conducted. For pairwise comparisons of prevalence estimates between questioning technique groups (e.g., π_ECWM_, π_DQ_), we assessed the difference in model fit (Δ*G*^*2*^) between an unrestricted baseline model in which the respective parameters could be estimated freely and a restricted alternative model in which the parameters were set equal (e.g., π_ECWM_ = π_DQ_). A significant difference in model fit indicated if a restriction was inadmissible because the respective parameters differed significantly from each other (e.g., π_ECWM_ ≠ π_DQ_).

## Results

## Prevalence of the sensitive attributes

**Fig. 2:**
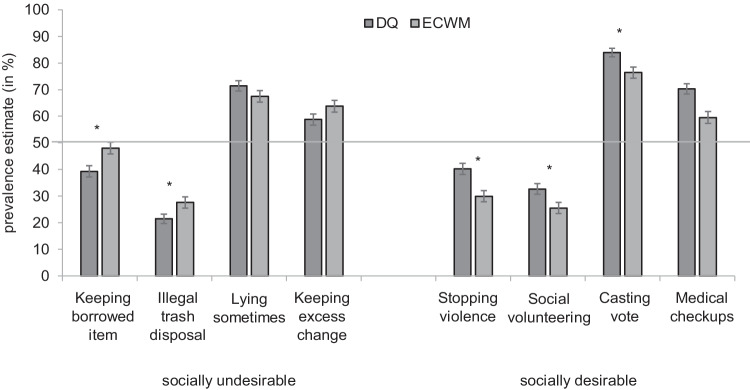
Prevalence estimates for sensitive attributes obtained via direct questions (DQ) and the Extended Crosswise Model (ECWM). *Error bars* represent standard errors. *Asterisks* indicate statistical significance (*p* < .05). For socially undesirable attributes *(left panel*), higher estimates in the ECWM compared to the DQ condition indicate a successful control of social desirability bias; for socially desirable attributes (*right panel*), lower estimates in the ECWM compared to the DQ condition indicate a successful control of social desirability bias

Prevalence estimates for each condition are shown in Fig. 2. All prevalence estimates were consistent with their assignment to low- or high-prevalence attributes in the pilot study.

### Socially undesirable, low prevalence

Estimates of the prevalence of not having returned a borrowed item was significantly higher in the ECWM condition ($\hat{\uppi}$ = 48.02%, *SE* = 2.23%) than in the DQ condition ($\hat{\uppi}$ = 39.27%, *SE* = 2.09%), Δ$\hat{\uppi}$ = 8.75%, Δ*G²* (1) = 8.14, *p* = .004. Estimates of the prevalence of having illegally disposed of trash were also significantly higher in the ECWM condition ($\hat{\uppi}$ = 27.59%, *SE* = 2.13%) than in the DQ condition ($\hat{\uppi}$ = 21.47%, *SE* = 1.76%), Δ$\hat{\uppi}$ = 6.12%, Δ*G²* (1) = 4.88, *p* = .027.

### Socially undesirable, high prevalence

Estimates of the prevalence of lying sometimes did not significantly differ between the ECWM condition ($\hat{\uppi}$ = 67.41%, *SE* = 2.16%) and the DQ condition ($\hat{\uppi}$ = 71.43%, *SE* = 1.92%), Δ$\hat{\uppi}$ = 4.02%, Δ*G²* (1) = 1.92, *p* = .166, but was descriptively higher in the DQ condition. Estimates of the prevalence of having kept too much change was descriptively higher in the ECWM condition ($\hat{\uppi}$ = 63.75%, *SE* = 2.19%) than in the DQ condition ($\hat{\uppi}$ = 58.77%, *SE* = 2.10%); this difference was however not significant, Δ$\hat{\uppi}$ = 4.98%, Δ*G²* (1) = 2.71, *p* = .099.

### Socially desirable, low prevalence

Estimates of the prevalence of stopping violence against women or children was significantly lower in the ECWM condition ($\hat{\uppi}$ = 29.97%, *SE* = 2.14%) than in the DQ condition ($\hat{\uppi}$ = 40.26%, *SE* = 2.09%), Δ$\hat{\uppi}$ = 10.26%, Δ*G²* (1) = 11.84, *p* < .001. Estimates of the prevalence of volunteering in the social sector was also significantly lower in the ECWM condition ($\hat{\uppi}$ = 25.50%, *SE* = 2.10%) than in the DQ condition ($\hat{\uppi}$ = 32.79%, *SE* = 2.01%), Δ$\hat{\uppi}$ = 7.20%, Δ*G²* (1) = 6.35, *p* = .012.

### Socially desirable, high prevalence

Estimates of the prevalence of having cast a vote in the last federal election was significantly lower in the ECWM condition ($\hat{\uppi}$ = 76.39%, *SE* = 2.07%) than in the DQ condition ($\hat{\uppi}$ = 83.95%, *SE* = 1.58%), Δ$\hat{\uppi}$ = 7.56%, Δ*G²* (1) = 8.32, *p* = .004. Estimates of the prevalence of going for regular medical checkups were also significantly lower in the ECWM condition ($\hat{\uppi}$ = 59.52%, *SE* = 2.20%) than in the DQ condition ($\hat{\uppi}$ = 70.30%, *SE* = 1.96%), Δ$\hat{\uppi}$ = 10.78%, Δ*G²* (1) = 13.13, *p* < .001.

## Model fits of the ECWM

The ECWM showed a good model fit for five of the eight attributes, indicating that no systematic answer bias had occurred (see Table 5). For three of the eight attributes (lying sometimes, having kept excess change, having cast a vote), the ECWM did not fit the data well. In the case of the ECWM, such misfits point towards the presence of a systematic answer bias towards one of the available answer options (Heck et al., 2018) that has to be distinguished from purely random answer behavior. In particular, we observed that for these three attributes, prevalence estimates in conditions with randomization probability *p2* were significantly lower than prevalence estimates in conditions with randomization probability *p1*. Specifically, the answer option “I agree with *exactly one* of the statements (irrespective of which one)” was chosen unexpectedly often in the *p2*-conditions, indicating a systematic preference for this answer option.

**Table 5:** Model fit and prevalence estimates for the ECWM groups with randomization probability p1 and p2 (standard errors in parentheses)

						*Model fit*		
Social Desirability	Prevalence (low / high)	Sensitive statement	${\hat{\pi}}_1$	${\hat{\pi}}_2$	Δ$\hat{\pi}$	Δ*G²* (1)		*p*
Socially undesirable	Low prevalence	I have not returned a borrowed item.	49.46 (3.15)	46.58 (3.16)	2.88	0.42	=	.520
		I have illegally disposed of trash.	26.63 (2.99)	28.55 (3.02)	1.92	0.20	=	.652
	High prevalence	I sometimes lie.	77.70 (2.91)	57.07 (3.14)	20.63	22.88	<	.001*
		I have kept change that was mistakenly given back to me in excess.	70.13 (3.02)	57.34 (3.14)	12.79	8.58	=	.003*
Socially desirable	Low prevalence	I have actively intervened to prevent violence against women or children.	27.60 (2.98)	32.37 (3.07)	4.77	1.25	=	.265
		I volunteer in the social sector.	25.99 (2.96)	25.00 (2.97)	0.99	0.06	=	.812
	High prevalence	I cast my vote in the last federal election.	86.48 (2.72)	66.24 (3.07)	20.24	24.00	<	.001*
		I go for regular medical checkups.	63.60 (3.09)	55.41 (3.14)	8.18	3.45	=	.063

Prevalence estimates ${\hat{\uppi}}_1$ correspond to the ECWM group with randomization probability *p1* (*p1* = .158) and prevalence estimates ${\hat{\uppi}}_2$ correspond to the ECWM group with randomization probability *p2* (*p2* = .842)

Following the advice of a reviewer, we performed additional analyses for the attributes for which we found a model misfit in the ECWM condition. To this end, we considered π1 and π2 as lower and upper bound estimates of the true prevalence and compared these estimates to the respective DQ estimates. For “lying sometimes”, we found that the lower bound estimate ($\hat{\uppi}$_ECWM_2_ = 57.07%, *SE* = 3.14%) was significantly lower than the DQ estimate ($\hat{\uppi}$_DQ_= 71.43%, *SE* = 1.92%), Δ*G²* (1) = 15.33, *p* < .001, whereas the upper bound estimate ($\hat{\uppi}$_ECWM_1_ = 77.70%, *SE* = 2.91%) did not differ significantly from the DQ estimate, Δ*G²* (1) = 3.19, *p* = .074. For “keeping excess change”, the lower bound estimate ($\hat{\uppi}$_ECWM_2_ = 57.34%, *SE* = 3.14%) did not significantly differ from the DQ estimate ($\hat{\uppi}$_DQ_= 58.77%, *SE* = 2.10%), Δ*G²* (1) = 0.14, *p* = .704, whereas the upper bound estimate ($\hat{\uppi}$_ECWM_1_ = 70.13%, *SE* = 3.02%) was significantly higher than the DQ estimate, Δ*G²* (1) = 9.39, *p* = .002. For “casting vote”, the lower bound estimate ($\hat{\uppi}$_ECWM_2_ = 66.24%, *SE* = 3.07%) was significantly lower than the DQ estimate ($\hat{\uppi}$_DQ_= 83.95%, *SE* = 1.58%), Δ*G²* (1) = 27.14, *p* < .001, whereas the upper bound ($\hat{\uppi}$_ECWM_2_ = 86.48%, *SE* = 2.72%) did not significantly differ from the DQ estimate, Δ*G²* (1) = 0.64, *p* = .423.

## Exploratory analyses: Additional variable on random responding

For exploratory purposes, we repeated the analyses for a small subsample in which respondents admitting a tendency to providing random answers (value of 2 or higher on the Likert scale; *n* = 5619, 86.39% of the total sample) were excluded. This did however not change the overall pattern of results. Prevalence estimates for the subgroup of respondents who did not indicate to have answered randomly can be found in Table A2 of the Appendix on osf (https://osf.io/jer5h/).

## Discussion

In many previous studies, the (E)CWM was found to lead to higher prevalence estimates than DQ for socially undesirable attributes with a prevalence below 50% and to lower prevalence estimates than DQ for socially desirable attributes with a prevalence above 50%. This pattern of results was often interpreted as evidence that the (E)CWM is successful in controlling social desirability bias. However, this pattern can also be the result of a methodological artifact when there is a substantial proportion of random responses. In the present study, we tested these two alternative explanations against each other by orthogonally manipulating both the direction of social desirability as well as the prevalence of the sensitive attributes, based on the framework suggested by Walzenbach and Hinz (2019). We found that—with one exception—for socially undesirable attributes ECWM estimates exceeded DQ estimates, and for socially desirable attributes ECWM estimates were lower than DQ estimates, irrespective of whether the prevalence of the sensitive attribute was above or below 50%. Six of the eight comparisons in the current study were statistically significant; however, in the condition with socially undesirable and highly prevalent attributes the comparisons of ECWM and DQ estimates remained insignificant.

The current study also assessed the model fit of the ECWM. For five of the eight attributes, the ECWM showed a good model fit, indicating that the prevalence estimates obtained were identical for both ECWM groups, and that the pooled estimates could therefore be considered trustworthy (Heck et al., 2018). For three of the eight attributes (lying sometimes, keeping excess change, casting vote), the ECWM did not fit the data well, indicating that the estimates differed between the two ECWM groups and that the pooled estimate was therefore potentially biased. For these attributes, we therefore performed additional analyses in which we considered estimates from the two ECWM groups as lower and upper bounds, respectively, and separately compared these estimates to the respective DQ estimates. For two of these attributes (keeping excess change, casting vote), one of the two bound estimates significantly differed from the respective DQ estimate in the direction indicating a successful control of social desirability bias rather than random responding. Specifically, the upper bound estimate for the socially undesirable attribute of keeping excess change was significantly higher than the DQ estimate; and the lower bound estimate for the socially desirable attribute of casting vote was significantly lower than the DQ estimate. In both cases, the other bound estimate (lower bound for keeping excess change and upper bound for casting vote) did not differ significantly from the DQ estimate. For these two attributes, a control of socially desirable responding thus seems to have been successful in only one of the two ECWM groups. This finding suggests that the respective attributes may not have been perceived as particularly sensitive by some of the respondents. Alternatively, this pattern might also point towards a potential link between perceived sensitivity and randomization probability. Differences between ECWM group estimates might also be due to systematic response biases resulting from lower comprehensibility or lower trust towards the method in one of the two ECWM groups. Importantly, however, the current results show that the two-group design of the ECWM clearly identifies such issues by indicating a model misfit.

For one attribute for which an ECWM misfit was observed (lying sometimes), the lower bound ECWM estimate was significantly lower than the DQ estimate. As “lying sometimes” is socially undesirable but highly prevalent, this difference cannot be explained by a successful control of social desirability bias; rather, it suggests that random responding biased the ECWM lower bound towards 50%. This finding points towards a potential lack of understanding or trust towards the method in one of the ECWM groups. Notably, recent results by Wolter and Diekmann (2021) suggest that high randomization probabilities in CWM questions (i.e., a high prevalence of the non-sensitive statement) are associated with biased estimates. This assumption is well compatible with the current findings because the lower bound estimate for “lying sometimes”, which was apparently influenced by random responding, was observed in the ECWM group with a high randomization probability (*p2*). However, it should be noted that neither the upper bound estimate nor the pooled ECWM estimate for lying sometimes differed significantly from the DQ estimate. Furthermore, and most importantly, the ECWM misfit indicated that the overall estimate should be interpreted with caution. This problem would have gone undetected in a single-group design employing the saturated CWM. In applied settings, the possibility to test the fit of a model is an important safeguard against the overinterpretation of prevalence estimates that may be subject to systematic biases.

Overall, the pattern of results found in the current study accords well with the notion that the ECWM generally helps to control for socially desirable responses. In contrast, if a substantial proportion of random responding had occurred, prevalence estimates in the ECWM condition should have been closer to 50% than prevalence estimates in the DQ condition, irrespective of whether attributes were socially undesirable or socially desirable. As no such pattern was observed, our results seem more consistent with the notion that the ECWM successfully controlled social desirability bias than with the notion that ECWM estimates were influenced by a substantial influence of random responding. Therefore, although based on the present results we cannot rule out a small proportion of random answers, random responding does not seem to be a primary, and certainly not the only, explanation for the findings of previous weak validation studies attesting to the improved validity of (E)CWM as compared with DQ estimates. Rather, the findings of the current study are in line with the results of previous strong validation studies which found that CWM estimates were significantly closer than DQ estimates to the known true prevalences of experimentally induced sensitive attributes (Hoffmann et al., 2015; Meisters et al., 2020a). Taken together with the results of the current study, these studies provide evidence supporting the notion that the CWM can contribute to the successful control of social desirability bias.

The validation approach used for the current study was superior to that of previous weak validation studies because we assessed attributes that were either socially undesirable or socially desirable and compared attributes that were either high or low in prevalence. We were, therefore, able to test and rule out random responses as a sufficient alternative explanation for the findings of previous weak validation studies that assessed only socially undesirable attributes with low prevalence, or socially desirable attributes with high prevalence.

There are some important advantages of the present “more- / less-is-better”-approach over strong validation studies: it is much less lavish than many strong validation designs, and it allows a test under more realistic conditions because it does not need to employ zero-prevalence attributes or slightly artificial cheating paradigms to induce sensitive attributes with known prevalence. In a previous study employing the CWM, only the prevalence of a socially desirable attribute with low prevalence was investigated (Walzenbach & Hinz, 2019). In this study, the CWM led to descriptively higher prevalence estimates than DQ. This finding stands in contrast to the findings of the present study; however, no test of the significance of the observed difference was reported. Moreover, only the present study allowed for a comprehensive assessment of the CWM’s validity since we implemented all four possible combinations of the direction of social desirability (socially undesirable vs. desirable) and prevalence (high vs. low). Our study thus included all four cells that are necessary to evaluate the competing accounts for the results of previous studies investigating the validity of the CWM. Moreover, we assessed two different attributes for each cell of the experimental design; that we observed similar outcomes for each of them lends further support to the robustness of our results.

Some previous studies found the CWM to produce problematic false positives under certain conditions (Höglinger & Diekmann, 2017; Höglinger & Jann, 2018; Meisters et al., 2020a). Random responding was discussed as a potential reason for the occurrence of false positives (Höglinger & Diekmann, 2017). The present study suggests that random responding is not a major problem when employing the CWM. So how can false positives be explained? First, an important difference between our study and some previous studies is that we did not investigate zero-prevalence attributes (as, e.g., in Höglinger & Diekmann, 2017). For such attributes, even small proportions of random responses necessarily lead to false positives. Such small proportions of random responses cannot be ruled out by our study; however, the effects of small proportions of random responses are less severe, and maybe even negligible, for attributes with a prevalence substantially higher than zero. Moreover, attributes with a prevalence of zero may be perceived as artificial by a part of the respondents, potentially leading to reactance and lower compliance with instructions, which may also contribute to a higher rate of false positives due to random responding.

In two of the previous studies that found false positives, experimentally induced sensitive attributes were investigated (Höglinger & Jann, 2018; Meisters et al., 2020a). The validity of strong validation studies based on experimentally induced sensitive attributes is called into question when self-deceptive respondents wrongly deny being a carrier of the sensitive attribute. Moreover, such studies can be criticized if they involve a deliberate deception of respondents or a secret recording of the respondents’ true status. The current study, in contrast, resembled realistic applications of the CWM much more closely, since it involved real sensitive attributes that were not generated for the experiment.

In summary, findings with regard to the validity of the CWM are mixed, with some studies reporting encouraging results and others indicating potential problems of the method. The exact reasons for the different outcomes have yet to be understood and are the subject of current scientific debate, but some potential candidates that have been identified in previous studies and the current work are: Sample characteristics, especially with regard to respondent education, since lower education has been linked to lower instruction comprehension (Meisters et al., 2020a); sample size, since estimates based on small samples are more susceptible to the influence of random error and response bias; mode of administration (e.g. online vs. offline; Sagoe et al., 2021); the exact wording of the sensitive statement under investigation, since the wording may cause attributes to be perceived as too sensitive, not sensitive enough, or ambiguous (Hoffmann et al., 2020; Jerke et al., 2022; Sagoe et al., 2021); the choice of the non-sensitive statement used for randomization and the respective randomization probability, since the current study has shown that different randomization probabilities can sometimes result in different prevalence estimates for the same sensitive attribute; and the number of groups used when employing the CWM or the ECWM as an indirect questioning technique, respectively. Most previous studies opted for the one-group design of the original CWM which only applies one randomization probability. Choosing the ECWM with two groups differing in their randomization probabilities allows to detect systematic response biases, and identify situations in which prevalence estimates might not be trustworthy, as indicated by model misfits. Such issues remain undetected when the original CWM with only one randomization probability is used.

## Limitations

A limitation of the current study is related to the misfit of the ECWM observed for three of the eight attributes under investigation. Prevalence estimates for these attributes should be interpreted with caution because there was evidence for a systematic response bias occurring when answering these questions. However, it is important to emphasize that the main conclusion of the current study remains unaffected even when only the remaining cells are considered. It is also interesting to note that model misfits occurred only for highly prevalent sensitive attributes. This raises the question of whether highly prevalent sensitive attributes pose a general problem for the (E)CWM, and why this might be the case. We observed that for attributes for which a model misfit was observed, prevalence estimates in conditions with randomization probability *p2* were always significantly lower than prevalence estimates in conditions with randomization probability *p1*. Moreover, for these attributes, the answer option “I agree with *exactly one* of the statements (irrespective of which one)” was chosen unexpectedly often in the *p2*-conditions, indicating a systematic preference for this answer option. Possibly, respondents preferred this answer option because they perceived it as self-protective. However, in a recent study investigating whether respondents washed their hands thoroughly during the COVID-19 pandemic, the ECWM showed a good model fit for a sensitive attribute as highly prevalent as washing hands (78%; Mieth et al., 2021). Model misfits of the ECWM for sensitive attributes with high prevalence might therefore only occur under certain conditions which should be identified in future studies on the ECWM.

Two of the three attributes for which a model misfit occurred (lying sometimes and keeping excess change) were rated as only slightly socially undesirable in the pilot study and might therefore not have been optimal choices for the present investigation. Future studies should seek to assess attributes that are more clearly socially desirable or undesirable.

## Conclusions

The current study found that differences between (E)CWM and DQ prevalence estimates can presumably better be accounted for by a successful control of socially desirable responding than by a substantial influence of random responses. Random responding does not seem to be a sufficient explanation for previous positive evaluations of the (E)CWM in weak and strong validation studies. Although the current study cannot rule out that there is a small influence of random responding on (E)CWM estimates, our results are more consistent with the notion that the (E)CWM provides more valid estimates than DQ. Our findings further suggest that for the specific case of socially undesirable attributes with high prevalence, (E)CWM estimates could potentially be biased and should be interpreted with caution, especially when high randomization probabilities are employed. However, such attributes are comparatively rare, and response biases associated with specific randomization probabilities can demonstrably be identified by assessing model fit using the two-group ECWM design. Taking these limitations and potential solutions into account, our results generally support the positive conclusions drawn regarding the (E)CWM’s validity in previous validation studies. When assessing the merits of the (E)CWM, it is also important to bear in mind that due to our very large sample size (*N* = 6504), the power to detect even small misfits and differences in prevalence estimates between ECWM conditions was very high.
